# Awareness, attitudes, and practice intentions toward telemedicine among Egyptian medical students and interns

**DOI:** 10.1186/s12909-026-09866-5

**Published:** 2026-07-13

**Authors:** Mayada Mohamed, Nada A. Albltagy, Israa Magdy Ata, Mai Ali Sabry, Maghfera Mohamed Abdelazeem, Mahmoud Magdy Zidan, Ammar Mohamed Abd-Elaziz, Ahmed Khairy Soliman, Asim K. Al-Husseini, Omar K. Badawy, Ahmed Hassan, Mohamed Basyouni Helal

**Affiliations:** 1 Faculty of Medicine, Suez University, Suez, Egypt; 2 Faculty of Medicine, Menoufia University, Menoufia, Egypt; 3 Faculty of Medicine, Mansoura University, Mansoura, Egypt; 4 Faculty of Medicine, Suez Canal University, Suez Canal, Egypt; 5 Faculty of Medicine, Ain Shams University, Cairo, Egypt; 6 Surgical Oncology Lecturer, Faculty of Medicine, Mansoura University, Mansoura, Egypt

**Keywords:** Telemedicine, Medical education, Knowledge, Attitude, Practice

## Abstract

**Background:**

Telemedicine offers a variety of medical services and facilitates the delivery of care, particularly in developing countries. In Egypt, there is limited evidence regarding the knowledge, attitudes, and perceptions of medical students and interns, representing a key segment of the future healthcare workforce.

**Objective:**

We aimed to assess awareness, attitudes, and practice intentions toward telemedicine among Egyptian medical students and interns, and to identify factors associated with positive attitudes toward its future clinical use.

**Methods:**

A multicenter cross-sectional study was conducted across 16 Egyptian medical universities using a structured questionnaire developed from previous studies and assessed for content and face validity. Proportionate quota sampling was applied, and multivariable logistic regression was used to identify factors associated with positive attitudes.

**Results:**

A total of 900 respondents were included in the analysis (51% males; mean age 21 ± 2 years). Overall, 556 (62%) were aware of telemedicine, among whom 76.3% demonstrated a positive attitude. High levels of internet access (89.2%) and digital comfort (83.9%) were reported; however, only 25% had prior telemedicine experience, and 19% attended related training. In multivariable analysis, digital comfort (AOR = 1.93; 95% CI: 1.06–3.49; *p* = 0.029) and prior telemedicine experience (AOR = 2.19; 95% CI: 1.35–3.55; *p* = 0.001) were significant positive predictors, while residence in Upper Egypt was negatively associated (AOR = 0.19; 95% CI: 0.08–0.44; *p* < 0.001).

**Conclusion:**

Despite generally favorable attitudes toward telemedicine, important disparities persist in awareness, exposure, and practical readiness among Egyptian medical students and interns. The observed awareness-practice gaps highlight the need for structured telemedicine education, digital health training, and competency-based integration within undergraduate medical curricula.

**Supplementary Information:**

The online version contains supplementary material available at 10.1186/s12909-026-09866-5.

## Introduction

Telemedicine refers to the remote delivery of healthcare services through electronic and web-based platforms, including teleconsultation, telediagnosis, telemonitoring, teletherapy, teleeducation, and social telephony [1]. Its role expanded substantially during the COVID-19 pandemic, when digital health became an essential component of healthcare delivery and medical education [2]. In developing countries, telemedicine may help address persistent barriers to healthcare access, including low physician-to-population ratios, limited specialist availability, and geographic isolation among rural and elderly populations. As telemedicine continues to expand globally, integrating it into medical curricula has become increasingly important to prepare future physicians for digitally enabled healthcare systems [1, 3].

Existing evidence has highlighted the growing importance of telemedicine and digital health education among healthcare students and professionals. For instance, a mixed-methods review by Waseh and Dicker (2019) demonstrated an increasing interest in integrating telemedicine into undergraduate medical education [4]. A scoping review by Xie et al. (2026) emphasized the need for structured digital health competencies within medical curricula [5]. Additionally, Car et al. (2025) provided an international consensus statement based on a Delphi study guided framework to incorporate telehealth among medical students and future physicians [6]. However, evidence from low- and middle-income countries, particularly in the Middle East and North Africa region, remains limited and heterogeneous.

Despite the potential benefits of telemedicine, medical students’ knowledge and perceptions vary considerably across different settings. In the United States, a study at Harvard Medical School reported that only 15.1% of students were knowledgeable about telemedicine before curriculum implementation [7]. Similar findings have been observed in developing countries. For instance, medical students in Pakistan reported lower familiarity and knowledge compared to clinicians [8]. Additionally, in Northern Iran and the United Arab Emirates, attitudes toward telemedicine were generally positive, and most students supported its inclusion in medical curricula [2, 9]. Nevertheless, many previous studies used convenience-based cross-sectional surveys, limiting generalizability, while contextual differences across healthcare systems restrict the applicability of these findings to Egyptian medical education.

The present study was informed by the Technology Acceptance Model (TAM), a widely recognized model for explaining technology adoption. TAM proposes that users’ acceptance of a technology is influenced primarily by perceived usefulness and perceived ease of use, which may shape attitudes and future intention to use the technology [10]. However, the current study was not designed to formally test TAM, develop TAM-based hypotheses, or operationalize its constructs as separate validated domains. Instead, TAM was used only as an interpretive lens to contextualize medical students’ and interns’ awareness, attitudes, and perceptions toward telemedicine within the broader literature on digital health acceptance.

In Egypt, telemedicine integration within healthcare systems and medical education remains at an early stage. Existing studies conducted in Egypt have primarily focused on physicians, demonstrating variability in knowledge and utilization across specialties and institutions [11–14]. In contrast, evidence regarding telemedicine awareness, attitudes, and perceptions among Egyptian medical students and interns remains scarce. Assessing the readiness of this group is important for guiding telemedicine integration into health professions education, promoting lifelong learning, enhancing patient safety, and understanding how perceptions may translate into future clinical practice. Therefore, this study aimed to assess awareness, attitudes, and perceptions toward telemedicine among Egyptian medical students and interns and to identify factors associated with positive attitudes toward its future clinical use. The findings may inform telemedicine curriculum development and support the preparation of a digitally competent future healthcare workforce in Egypt.

## Methods

### Study design and setting

We conducted a multicenter cross-sectional study between August 2025 and December 2025 across 16 Egyptian medical universities (public and private) representing four geographic regions: Cairo and Alexandria (Kasr Al-Ainy, Ain Shams, MTI, MUST, Helwan, and Alexandria University), the Delta region (Kafr El-Sheikh, Mansoura, Menoufia, Tanta, and Zagazig University), the Suez Canal region (Suez, Suez Canal, and Port Said University), and Upper Egypt (Sohag and Assiut Universities).

### Study participants and sampling

Eligible participants included Egyptian undergraduate medical students and interns aged ≥ 18 years who were currently enrolled at one of the selected universities and were willing to provide informed consent. Non-Egyptian students were excluded from the study.

The sample size was calculated using Cochran’s formula [15] with a 95% confidence level, a 5% margin of error, and an estimated prevalence of 59% for adequate telemedicine knowledge, based on a previous nationwide study conducted among medical students in Pakistan [16]. The minimum required sample size was 372 participants. The final target sample size was inflated to 900 participants to increase the statistical power and ensure adequate representation of small-sized universities.

A proportionate non-random quota sampling strategy was applied to ensure balanced representation across universities. However, no formal student rosters (i.e., centralized lists with contact information) were available to the research team. Therefore, participants were recruited through university-affiliated social media groups (e.g., official Facebook, Telegram, and WhatsApp groups for each medical faculty).

Participants were recruited from an estimated total population of 105,368 medical students and interns across the included institutions. Each university was allocated a quota proportional to its estimated number of medical students and interns combined at that university using this equation: (Sample size per university = (Total number of medical trainees in the university / Total population across all universities) × Total sample size (*n* = 900)). Detailed quota allocations for all participating universities are provided in Supplementary File 1. The survey link was distributed broadly within each institution until the target quota for that university was reached. Consequently, individual response rates per university could not be calculated.

### Questionnaire development

A structured, self-administered online questionnaire was developed following a comprehensive review of the literature addressing telemedicine awareness, attitudes, and practices among medical trainees. The questionnaire was designed to comprehensively assess participants’ demographic characteristics, digital access and literacy, awareness of telemedicine, attitudes toward its implementation, and current practices and future intentions.

The first section collected sociodemographic information, including age, sex, academic level, and university affiliation. The second section evaluated participants’ access to computers and the internet, reliability of connectivity, and their self-reported comfort in using digital technologies. Comfort was assessed using a 5-point scale ranging from 1 (strongly uncomfortable) to 5 (strongly comfortable). The awareness section explored whether participants had previously heard of telemedicine, had any prior experience using telemedicine services, or had attended lectures, courses, or training sessions related to telemedicine. Only participants who reported prior awareness of telemedicine (62%, *n* = 556) were directed to complete the subsequent attitude and practice sections through built-in questionnaire branching logic, whereas unaware participants (38%, *n* = 344) automatically bypassed these items. Consequently, all descriptive and inferential evaluations of attitudes and practices are strictly restricted to this baseline-aware sub-population rather than the full sample (*n* = 900).

Attitudes toward telemedicine were assessed using seven statements rated on a 5-point Likert scale (1 = strongly disagree to 5 = strongly agree). These statements addressed perceived effectiveness, future relevance, impact on clinical practice, usefulness in chronic disease monitoring, accessibility for remote patients, encouragement of implementation in Egypt, and potential threats to traditional medical practice.

The practice section evaluated participants’ preference to continue using some form of telemedicine in clinical practice, confidentiality concerns compared with face-to-face consultations, intentions to incorporate telemedicine into future practice, and support for including telemedicine training in the medical curriculum.

Content and face validity of the questionnaire were evaluated by two experts in medical education and digital health. A pilot study was conducted on 30 students from the target population to assess clarity, feasibility, comprehensibility, and reliability. Internal consistency for the attitude scale was acceptable (Cronbach’s α = 0.726). Pilot participants did not participate in the final data collection. Minor wording modifications were made based on feedback obtained during pilot testing. A sample of the questionnaire and scoring instructions are provided in Supplementary File 2.

### Data collection

An online self-administered Google Form was distributed through university-affiliated social media groups (e.g., official Facebook, Telegram, and WhatsApp groups for each medical faculty) across the participating universities. Electronic informed consent was obtained from all participants prior to questionnaire completion. Participation was voluntary, and responses were collected anonymously. To minimize duplicate submissions, the survey settings were configured to permit only one response per registered email account.

### Ethics considerations

Ethical approval was obtained from the Research Ethics Committee at Mansoura University (ID: R.25.09.3363.R2–20251119). The study was conducted in accordance with the principles of the Declaration of Helsinki [17]. Participation was voluntary, and electronic informed consent was obtained from all participants. All responses were collected anonymously and kept confidential.

### Statistical analysis

Data were analyzed using IBM SPSS Statistics version 27.0 (IBM Corp., Armonk, NY, USA). Categorical variables were summarized as frequencies and percentages, while continuous variables were expressed as means and standard deviations or medians and interquartile ranges, as appropriate. Attitudes toward telemedicine were assessed using seven items rated on a 5-point Likert scale (1 = strongly disagree to 5 = strongly agree), yielding a total score ranging from 7 to 35. Items 1–6 were coded positively; item 7 (“Telemedicine is a threat to current medical practice”) was reverse-coded. The sum was calculated, and participants with total scores ≥ 60% of the maximum possible score were classified as having a positive attitude toward telemedicine, while those scoring < 60% were classified as having a negative attitude. The 60% cutoff was based on Bloom’s cutoff criteria [18, 19], which have been used in previous KAP studies to categorize scores as sufficient when ≥ 60% and insufficient when < 60%. To reduce the limitations of dichotomization, we additionally reported continuous attitude scores, including mean, standard deviation, median, and interquartile range, alongside the categorical classification.

Univariable binary logistic regression analyses were performed to identify factors associated with positive attitudes. Variables with a p-value < 0.20 in univariable analysis were entered into a multivariable logistic regression model to adjust for potential confounders. Results were reported as odds ratios (ORs) and adjusted odds ratios (aORs) with 95% confidence intervals (CIs). Model performance was evaluated using the Omnibus test of model coefficients and the Hosmer–Lemeshow goodness-of-fit test. A two-sided p-value < 0.05 was considered statistically significant.

## Results

### Demographic data of the participants

**Fig. 1:**
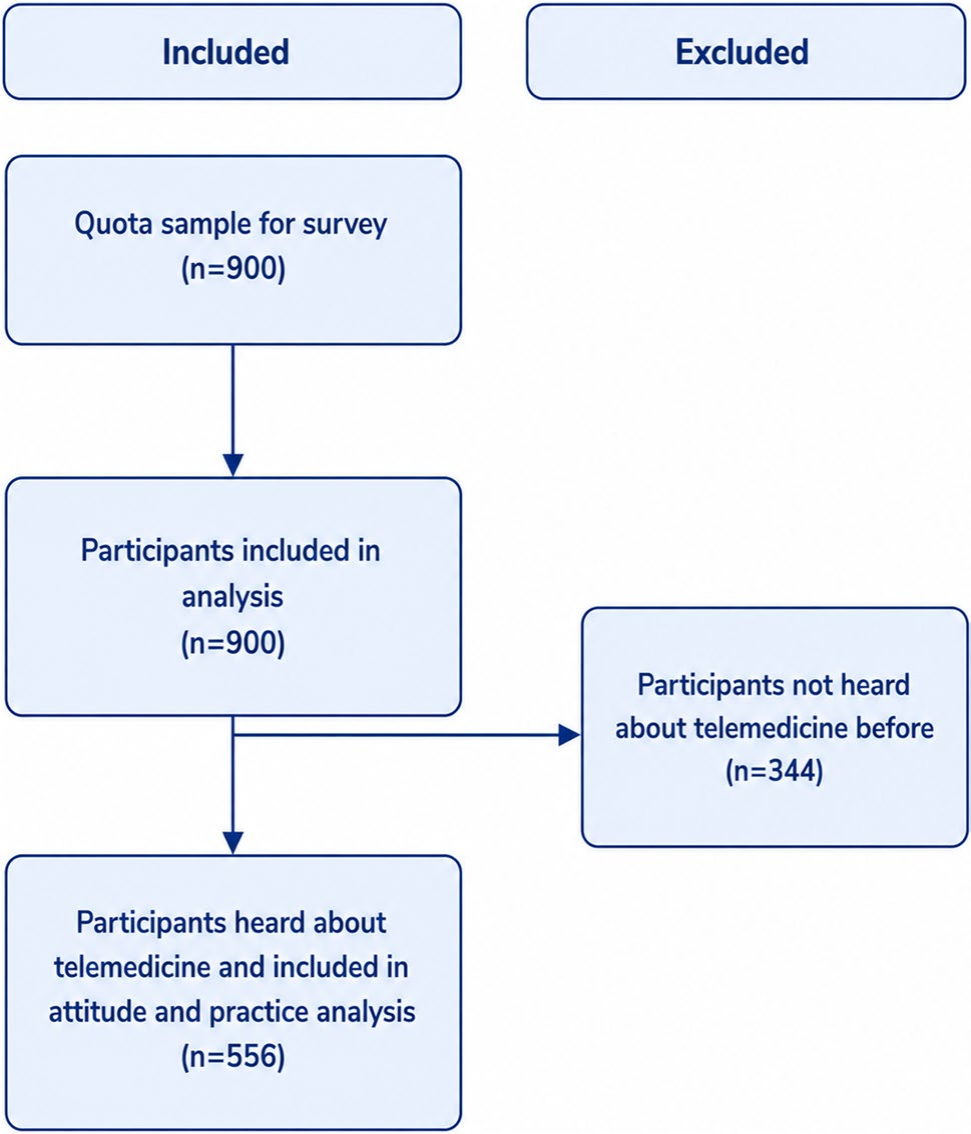
Flow diagram of participant selection and inclusion in the study

Table 1 presents the demographic characteristics of the study participants. A total of 900 individuals completed the questionnaire and were included in the final analysis (Fig. 1). Of the analyzed sample, 459 (51%) were male, and the mean age was 21 ± 2 years. Regarding academic level, 605 participants (67.2%) were in their clinical years, followed by 196 (21.8%) in preclinical years and 99 (11%) interns. In terms of geographic distribution, 428 participants (47.6%) were from the Cairo and Alexandria region, 337 (37.4%) from the Delta region, 56 (6.2%) from the Suez Canal region, and 79 (8.8%) from Upper Egypt.

**Table 1:** Demographic Characteristics of the Participants (*N* = 900)

Variable	Value
Age (years), mean ± SD	21 ± 2
Sex	
− Male	459 (51.0%)
− Female	441 (49.0%)
Academic year*	
− Preclinical	196 (21.8%)
− Clinical	605 (67.2%)
− Intern	99 (11.0%)
Geographic region	
− Cairo and Alexandria region	428 (47.6%)
− Delta region	337 (37.4%)
− Suez Canal region	56 (6.2%)
− Upper Egypt	79 (8.8%)

* Preclinical years (1st and 2nd year) and clinical years (3rd year to 5th year) were collapsed and categorized according to curricular structure

### Internet access and awareness of telemedicine

As shown in Table 2, 803 participants (89.2%) reported easy access to the internet and digital devices at home. Additionally, 755 (83.9%) reported being comfortable using digital devices, with a mean comfort score of 3 ± 1.2 on a 5-point Likert scale, with scores ranging from 1 to 5. Regarding awareness, 556 participants (62%) reported having heard about telemedicine. However, only 222 (25%) reported prior use or experience with telemedicine, and 174 (19%) attended lectures or courses related to telemedicine.

**Table 2:** Internet Access and Awareness of Telemedicine (*N* = 900)

Variables	Yes, *n* (%)	No, *n* (%)
Internet access and digital device use		
Easy access to the internet and digital devices at home	803 (89.2%)	97 (10.8%)
Comfortable using internet or digital devices	755 (83.9%)	145 (16.1%)
Mean score for comfort using internet or digital devices (1–5)	3 ± 1.2	
Awareness		
Ever heard about telemedicine	556 (62%)	344 (38%)
Prior use or experience	222 (25%)	678 (75%)
Attended telemedicine lectures or courses	174 (19%)	726 (81%)

Mean comfort score was calculated using a 5-point scale from 1 to 5, where 1 = strongly uncomfortable, 5 = strongly comfortable)

### Attitudes and practices toward telemedicine

As shown in Table 3, only the 556 participants who reported prior awareness of telemedicine completed the attitude and practice sections. The mean overall attitude score among aware participants was 25.92 ± 4.01 (median = 26; IQR: 24–28). Based on the predefined cutoff (≥ 60% ), 424 participants (76.3%) were classified as having a positive attitude toward telemedicine.

**Table 3:** Attitudes and Practices Toward Telemedicine (N=556)

Table 3A: Attitudes Toward Telemedicine						
Variables	Strongly agree *n* (%)	Agree *n* (%)	Neutral *n* (%)	Disagree *n* (%)	Strongly disagree *n* (%)	Mean ± SD
Telemedicine is a major change in medicine.	103 (19%)	278 (50%)	140 (25%)	28 (5%)	7 (1%)	3.79 ± 0.84
Telemedicine will play an important role in future clinical practice.	132 (24%)	281 (51%)	108 (19%)	29 (5%)	6 (1%)	3.91 ± 0.85
Telemedicine reduces medical errors and saves time	70 (13%)	166 (30%)	165 (29%)	127 (23%)	28 (5%)	3.22 ± 1.08
Telemedicine is useful for monitoring chronic diseases.	177 (32%)	264 (48%)	83 (15%)	24 (4%)	8 (1%)	4.04 ± 0.87
Telemedicine improves access to healthcare, especially for rural populations.	191 (34%)	267 (48%)	65 (12%)	26 (5%)	7 (1%)	4.10 ± 0.86
The use of telemedicine should be encouraged.	109 (20%)	261 (47%)	139 (25%)	38 (7%)	9 (1%)	3.76 ± 0.90
Telemedicine is a threat to current medical practice.	41 (7%)	126 (23%)	162 (29%)	190 (34%)	37 (7%)	3.10 ± 1.05
Total attitude score Mean ± SD Median (IQR)	25.92 ± 4.01 26 (24–28)					
Attitude Categories Positive attitude Negative attitude	424 (76.3%) 132 (23.7%)					

Table 3B: **Practices Toward Telemedicine**					
**Variables**	**Strongly agree** **n (%)**	**Agree** **n (%)**	**Neutral** **n (%)**	**Disagree** **n (%)**	**Strongly disagree** **n (%)**
I prefer to continue using some form of telemedicine in clinical practice.	55 (10%)	195 (35%)	201 (36%)	81 (15%)	24 (4%)
Telemedicine poses a risk to patient confidentiality compared to face-to-face consultations.	35 (6%)	81 (15%)	101 (18%)	231 (42%)	108 (19%)
Medical curricula should include telemedicine education and training.	154 (28%)	268 (48%)	108 (19%)	18 (3%)	8 (2%)
Do you think telemedicine has an important role in diagnosis in the current practice?	Yes No Maybe	142 (26%) 145 (26%) 269 (48%)			
Would you like to incorporate telemedicine into your future practice as a physician?	Yes No Maybe	224 (40%) 93 (17%) 239 (43%)			

In Table 3B: the first three items were assessed using a 5-point scale from 1 to 5 (1 = strongly disagree, 5 = strongly agree), while the last 2 items were assessed using a Yes/No/Maybe scale. The item ‘Telemedicine is a threat to current medical practice’ was reverse-coded for calculation of the total attitude score.

Regarding perceived benefits, a majority of the aware participants agreed that telemedicine represents a major change in medicine (69%) and is important for the future of healthcare (75%). Additionally, 80% agreed that telemedicine facilitates monitoring of chronic conditions, and 82% agreed that it provides quicker access to care.

However, concerns regarding efficiency and risk were evident among aware participants. Only 43% agreed that telemedicine reduces medical errors and saves time. Furthermore, 30% agreed that telemedicine may negatively impact the medical field. With respect to future intentions and practices, 76% agreed that telemedicine-related lectures should be incorporated into medical education in Egypt. Nevertheless, fewer participants expressed readiness for direct implementation: 45% prefer to continue using some form of telemedicine in clinical practice, 40% expressed willingness to incorporate telemedicine into their future practice as physicians, 21% believed telemedicine poses a risk to patient confidentiality compared to face-to-face consultations, and 26% believed that telemedicine has an important role in diagnosis in current practice.

### Factors associated with positive attitude toward telemedicine

In univariable logistic regression analysis, younger age, easy internet access at home, comfort using digital devices, prior telemedicine experience, and attendance at telemedicine lectures were significantly associated with higher odds of a positive attitude (*p* < 0.05). In contrast, residence in Upper Egypt was associated with lower odds of a positive attitude. Variables with *p* < 0.20 in univariable analysis were entered into the multivariable logistic regression model.

**Fig. 2:**
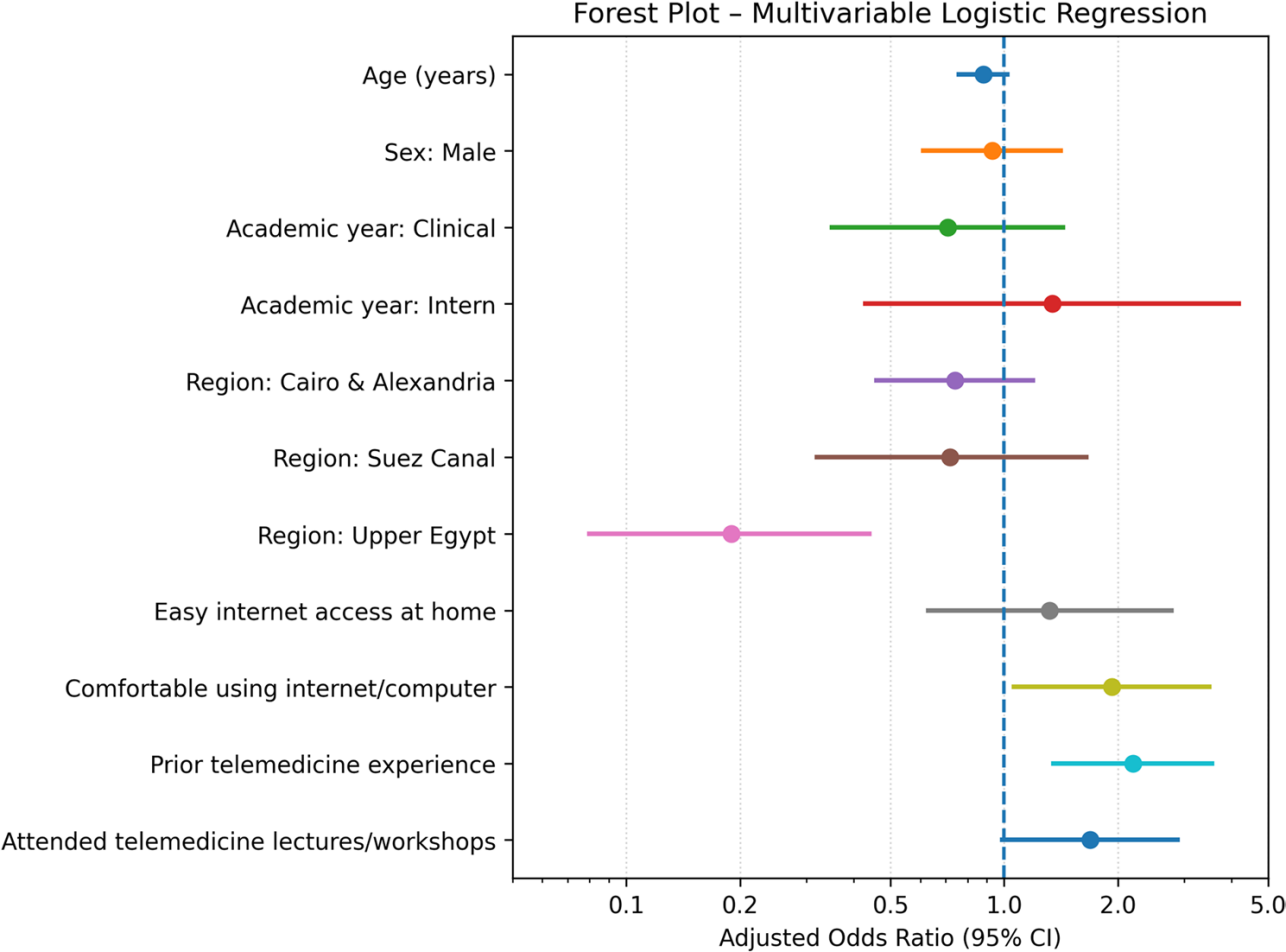
Forest plot of multivariable logistic regression analysis of factors associated with positive attitude toward telemedicine

In the multivariable logistic regression analysis, several factors remained independently associated with a positive attitude toward telemedicine. After adjustment f*or* potential confounders, comfort using the internet or computer was significantly associated with higher odds of a positive attitude (adjusted OR = 1.93, 95% CI: 1.06–3.49, *p* = 0.029). Similarly, prior use or experience with telemedicine remained a strong independent predictor (adjusted OR = 2.19, 95% CI: 1.35–3.55, *p* = 0.001). Conversely, students residing in Upper Egypt were independently associated with significantly lower odds of a positive attitude (adjusted OR = 0.19, 95% CI: 0.08–0.44, *p* < 0.001). Other variables, including age, sex, academic year, region (Cairo & Alexandria and Suez Canal), easy internet access at home, and attendance at telemedicine lectures or workshops, were not statistically significant in the adjusted model (*p* > 0.05) (Table 4; Fig. 2).

**Table 4:** Univariable and multivariable logistic regression analyses of factors associated with positive attitude regarding telemedicine

Variable	Univariable		Multivariable	
	OR (95% CI)	*P*-value	Adjusted OR (95% CI)	*P*-value
Age	0.88 (0.80–0.97)	0.012*	0.88 (0.76–1.02)	0.094
Sex = Male	1.40 (0.95–2.08)	0.092	0.93 (0.61–1.41)	0.755
Academic Year				
− Preclinical	Reference		Reference	
− Clinical	0.65 (0.42–1.02)	0.058	0.71 (0.35–1.43)	0.340
− Intern	1.07 (0.58–1.97)	0.837	1.34 (0.43–4.18)	0.608
Region				
− Delta region	Reference		Reference	
− Cairo & Alex region	1.25 (0.85–1.86)	0.262	0.74 (0.46–1.19)	0.224
− Suez Canal region	1.14 (0.51–2.55)	0.754	0.72 (0.32–1.65)	0.449
− Upper Egypt	0.22 (0.11–0.44)	< 0.001*	0.19 (0.08–0.44)	< 0.001*
Easy internet access at home = Yes	2.42 (1.28–4.57)	0.007*	1.32 (0.63–2.77)	0.452
Comfortable using the internet/computer = Yes	3.25 (1.93–5.46)	< 0.001*	1.93 (1.06–3.49)	0.029*
Prior use or experience with telemedicine = Yes	2.07 (1.34–3.19)	0.001*	2.19 (1.35–3.55)	0.001*
Attended telemedicine lectures or workshops = Yes	1.86 (1.16–3.00)	0.010*	1.69 (0.99–2.88)	0.053

Model performance: Omnibus χ²(11) = 60.52, p < 0.001; −2LL = 571.29; Cox & Snell R² = 0.103; Nagelkerke R² = 0.152; Hosmer–Lemeshow χ²(8) = 10.02, p = 0.264; overall accuracy = 76.1%

Bold values and asterisks indicate statistical significance at *p* < 0.05.

## Discussion

To our knowledge, this is one of the first multicenter studies assessing telemedicine readiness among medical students and interns in Egypt. In this study, a relatively high proportion of positive attitudes toward telemedicine was observed. Although 62% of participants reported awareness, only 25% had prior experience, highlighting a gap between awareness and practical exposure. This contrasts with higher awareness and utilization rates reported in studies conducted during and after the COVID-19 pandemic, when telemedicine use expanded rapidly, and familiarity increased among healthcare professionals [20–22]. The relatively lower exposure observed in our study suggests that telemedicine has not yet been systematically integrated into Egyptian medical training and clinical practice.

Among participants who were aware of telemedicine, 76.3% demonstrated a positive attitude, indicating strong acceptance despite limited hands-on experience. Most respondents perceived telemedicine as a transformative component of the healthcare system and emphasized its importance in future practice. These findings are consistent with prior studies conducted in low- and middle-income countries, which have reported similarly favorable perceptions among healthcare professionals and students [3, 23].

Notably, despite the favorable perceptions, our participants still had concerns about efficacy and risks, with 30% believing telemedicine could harm the medical field. However, a larger proportion disagreed, suggesting that telemedicine is more commonly viewed as a complementary tool rather than a replacement for conventional care. This diversity in perceptions aligns with findings from Libya, where healthcare workers expressed acceptance of telemedicine alongside concerns about its implications for physician roles, patient-doctor interaction, and professional identity [24]. The persistence of neutral or cautious responses likely reflects limited practical exposure and uncertainty regarding long-term integration into routine clinical workflows.

The predominance of positive attitudes observed in this study did not translate into corresponding practice intentions, indicating a clear awareness–practice intention gap. Only 21% of participants agreed or strongly agreed that telemedicine poses a risk to patient confidentiality compared with face-to-face consultations, and confidence in the accuracy of remote diagnosis remained low (26%), reflecting limited trust in telemedicine’s clinical reliability. In Egypt, there are continuous proactive measures by the government and healthcare providers to identify barriers to telemedicine adoption [25]. Despite our focus on future healthcare providers, students, and interns, our findings align with similar Egyptian studies on physicians. For instance, Alboraie et al. (2021) reported similar concerns regarding patients’ safety and privacy, as well as fear of higher medical errors [11]. Younis et al. (2025) found that barriers to telemedicine included a lack of training and education, poor infrastructure, and technical difficulty [14]. Regional diversity and resource availability across different settings were also identified as barriers. This underscores the need for policy development to improve training and educational curricula, as well as infrastructure [11, 25].

Additionally, similar concerns were reported among international medical students and trainees regarding confidentiality, accessibility, and diagnostic accuracy, particularly in the absence of structured telemedicine education [26]. Further international evidence suggests that concerns about diagnostic accuracy, reduced physical examination, and weak patient–physician rapport remain significant barriers to the acceptance of telemedicine, particularly among trainees [27, 28].

On the other hand, substantial global evidence supports the effectiveness of telemedicine in enhancing access, continuity, and timeliness of care when implemented appropriately [3, 29, 30]. Additionally, a diagnostic study conducted in a multispecialty healthcare institution in the US revealed that professional diagnoses by video telemedicine matched those made face-to-face in 86.9% of cases [31]. Furthermore, a systematic review concluded that teleconsultations are an effective alternative to face-to-face consultations, especially in primary care and mental health [29]. This discrepancy between established evidence and participants’ concerns highlights a critical knowledge gap, emphasizing the need for integrating structured telemedicine training into medical curricula to improve confidence, correct misconceptions, and facilitate its effective adoption in clinical practice.

Regarding future clinical practice intentions, participants expressed cautious openness. While a considerable proportion anticipated that telemedicine would be part of future medical practice, uncertainty remained common, and fewer than half expressed willingness to accept remote consultations. Similar concerns have been reported in Middle Eastern countries, including cultural, financial, organizational, technological, and regulatory challenges [32]. Additionally, in Pakistan, infrastructural limitations, regulatory uncertainty, and insufficient training have been identified as major obstacles to adoption [33]. Importantly, outright rejection of telemedicine was relatively limited, suggesting that targeted educational and system-level interventions may be associated with lower resistance.

When interpreted through TAM, the positive attitudes observed among students and interns may reflect perceived usefulness and anticipated relevance to future clinical practice. However, the gap between favorable attitudes and limited willingness to rely on remote consultations suggests that acceptance may still be affected by concerns about clinical reliability, confidentiality, and limited practical exposure. Since TAM constructs were not formally measured or modeled, this interpretation remains exploratory. Future studies should use validated TAM-based tools to assess perceived usefulness, perceived ease of use, attitudes, and intention to use telemedicine among Egyptian medical trainees.

Notably, educational preparedness emerged as one of the most significant gaps identified in our study. Despite strong support for the introduction of telemedicine-focused lectures, only a small proportion of participants attended formal courses or received structured training. This educational deficit aligns with findings from studies conducted across Africa and South Asia, where a lack of formal instruction has consistently been identified as a key barrier to telemedicine implementation [33–35]. Another study revealed that structured curricula were associated with improved knowledge, self-efficacy, and competency after completing formal training programs [7]. The strong support for curricular integration observed in this study reinforces the need to incorporate telemedicine competencies into undergraduate medical education to ensure future physicians are adequately prepared for digital healthcare delivery.

The multivariable regression analysis provided important insights into determinants of positive attitudes toward telemedicine. Digital comfort and prior telemedicine experience were the only independent positive predictors, highlighting the critical roles of digital literacy and experiential learning in shaping attitudes. In contrast, residence in Upper Egypt was independently associated with lower odds of a positive attitude, likely reflecting regional disparities in digital infrastructure, healthcare access, and resource availability. Other factors, including age, sex, and academic level, were not significant after adjustment. This suggests that perception toward telemedicine is associated with modifiable factors (e.g., exposure and digital competence) rather than demographics being intrinsic characteristics [36]. These findings are consistent with previous research demonstrating that infrastructural and regional inequalities remain major barriers to equitable telemedicine implementation in under-resourced settings [14, 34, 37].

Despite reliable internet access and perceived digital literacy, concerns regarding data privacy and security persisted. This aligns with international literature identifying confidentiality, data protection, and trust as recurring challenges to telemedicine adoption [26, 38]. Therefore, addressing these concerns through clear regulatory frameworks, ethical guidelines, and robust technical safeguards will be essential to build trust and enhance acceptance among future healthcare providers.

In summary, Egyptian medical students and interns generally demonstrated positive attitudes toward telemedicine, accompanied by significant educational, experiential, and infrastructural gaps. These findings suggest the value of structured telemedicine education, expanded clinical exposure, and targeted policies to address regional disparities. Strengthening these areas may be associated with more confident adoption of telemedicine and support its sustainable integration into Egypt’s healthcare system.

This study demonstrates several notable strengths. The inclusion of 16 universities across different geographic regions enhances the representativeness and generalizability of medical students and interns. Additionally, the inclusion of both medical students and interns captures perspectives across different stages of training, providing a more comprehensive assessment of telemedicine readiness. Furthermore, the diversity of institutions (public and private) and participant backgrounds (urban and rural) strengthens the external validity of the findings.

Despite the strengths, our study had some limitations. This cross-sectional study could not establish causal inferences, and self-reported data may be subject to bias. Although data were collected from both undergraduate medical students and interns, the findings cannot be generalized to all medical trainees, as differences in curricula, institutional resources, and exposure to telemedicine may influence the results. Selection bias due to quota-based online recruitment may have overestimated digital literacy among our participants, as students comfortable with digital tools may have been more likely to complete the online survey. Several practice and perception variables were single items, whose reliability and validity could not be assessed. The regression model explains only about 10–15% of the variance, indicating that other unmeasured variables, such as institutional support or personality traits, could have affected attitudes. Finally, given the rapid pace of digital health development, the study’s conclusions may be time-limited, as telemedicine tools and educational practices continue to evolve rapidly.

Based on our findings, enhancing telemedicine education and implementation in Egypt requires a multifaceted approach. It is essential to integrate telemedicine into medical curricula, either as mandatory or elective courses, with a focus on practical competencies, legal and ethical considerations, and simulated clinical training. In parallel, strengthening digital infrastructure, including reliable internet connectivity and access to appropriate devices in universities and teaching hospitals, is crucial to support effective training. Developing clear national and institutional guidelines, accompanied by targeted training on data protection, confidentiality, and cybersecurity, is also necessary to build trust and ensure safe implementation. Moreover, encouraging student and faculty research on telemedicine within the Egyptian context, along with organizing national seminars and conferences in collaboration with health authorities, may further promote adoption and optimize its integration into healthcare practice. Further longitudinal studies are required to assess the long-term impacts of educational training curricula on the improvement of attitudes and clinical preparedness.

## Conclusion

In conclusion, this study identified a gap between favorable perceptions of telemedicine and concerns regarding safety and efficacy. Addressing this gap requires structured training and educational curricula to prepare future healthcare workers to integrate telemedicine into their clinical training, ensuring that future physicians are well-prepared to utilize this evolving technology for better patient care in Egypt.

## Supplementary Information

Supplementary Material 1.

Supplementary Material 2.

## Data Availability

The datasets generated and analyzed during the current study are not publicly available due to participant privacy and confidentiality considerations but are available from the corresponding author on reasonable request.
