# Supplementary material for: Awareness, attitudes, and practice intentions toward telemedicine among Egyptian medical students and interns
Source: BMC Med Educ. 2026 Jul 13;26:1141. doi: 10.1186/s12909-026-09866-5 (PMC13366818; doi:10.1186/s12909-026-09866-5)
Supplement: Supplementary file 1 — Supplementary Material 1. [file 12909_2026_9866_MOESM1_ESM.docx]

**Supplementary Table S1: Population Distribution and Proportional Quota Allocation Across Participating Universities**

| University | Population | Proportion (%) | Sample (out of 900) |
| --- | --- | --- | --- |
| Suez University | 1277 | 1.2% | 11 |
| Suez Canal University | 2970 | 2.8% | 25 |
| Ain Shams | 9692 | 9.2% | 83 |
| Menoufia | 7000 | 6.6% | 59 |
| Mansoura | 9711 | 9.2% | 83 |
| Zagazig | 10000 | 9.5% | 86 |
| Tanta | 7300 | 6.9% | 62 |
| Alexandria | 15242 | 14.5% | 130 |
| Kafr El Sheikh | 4918 | 4.7% | 42 |
| MTI | 3099 | 2.9% | 26 |
| MUST | 6000 | 5.7% | 51 |
| Sohag | 3465 | 3.3% | 30 |
| Helwan | 4481 | 4.3% | 39 |
| Assiut | 5651 | 5.4% | 49 |
| Port Said | 2068 | 2% | 18 |
| Cairo (Kasr Al Ainy) | 12494 | 11.8% | 106 |
| Total | 105368 | 100% | 900 |
