# Supplementary material for: Awareness, attitudes, and practice intentions toward telemedicine among Egyptian medical students and interns
Source: BMC Med Educ. 2026 Jul 13;26:1141. doi: 10.1186/s12909-026-09866-5 (PMC13366818; doi:10.1186/s12909-026-09866-5)
Supplement: Supplementary file 2 — Supplementary Material 2. [file 12909_2026_9866_MOESM2_ESM.docx]

**Study Questionnaire**

**Section 1: Sociodemographic Characteristics**

1. **Age:** ______
2. **Sex:**
   - Male
   - Female
3. **Academic Level:**
   - 1st Year
   - 2nd Year
   - 3rd Year
   - 4th Year
   - 5th Year
   - Intern
4. **University:**
   - Ain Shams University
   - MTI University
   - Tanta University
   - MUST University
   - Cairo University (Kasr El-Ainy)
   - Kafr El-Sheikh University
   - Alexandria University
   - Mansoura University
   - Menoufia University
   - Suez University
   - Suez Canal University
   - Zagazig University
   - Helwan University
   - Port Said University
   - Sohag University
   - Assiut University

**Section 2: Internet access and digital device use**

1. Do you have easy access to the internet and digital devices at home?
   - Yes
   - No
2. Do you feel comfortable using the internet or digital devices?
   - Yes
   - No
3. On a scale from 1 to 5 (1 = strongly uncomfortable, 5 = strongly comfortable), how comfortable are you using personal information through communication technology?
   - 1
   - 2
   - 3
   - 4
   - 5

**Section 3: Awareness of Telemedicine**

1. Have you ever heard of telemedicine?
   - Yes
   - No
2. Have you ever had personal experience with telemedicine?
   - Yes
   - No
3. Have you ever attended lectures or courses related to telemedicine?
   - Yes
   - No

**Section 4: Attitudes Toward Telemedicine**

(5-point Likert scale: Strongly agree – Agree – Neutral – Disagree – Strongly disagree)

1. Telemedicine is a major change in medicine.
2. Telemedicine will play an important role in future clinical practice.
3. Telemedicine reduces medical errors and saves time
4. Telemedicine is useful for monitoring chronic diseases.
5. Telemedicine improves access to healthcare, especially for rural populations.
6. The use of telemedicine should be encouraged.
7. Telemedicine is a threat to current medical practice.

**Section 5: Perceptions Toward Telemedicine**

1. I prefer to continue using some form of telemedicine in clinical practice. (Likert scale: Strongly agree – Agree – Neutral – Disagree – Strongly disagree)
2. Telemedicine poses a risk to patient confidentiality compared to face-to-face consultations. (Likert scale: Strongly agree – Agree – Neutral – Disagree – Strongly disagree)
3. Medical curricula should include telemedicine education and training. (Likert scale: Strongly agree – Agree – Neutral – Disagree – Strongly disagree)
4. Do you think telemedicine has an important role in diagnosis in current practice?
   - Yes
   - No
   - Maybe
5. Would you like to incorporate telemedicine into your future practice as a physician?
   - Yes
   - No
   - Maybe

**Scoring Instructions**

Section 4: Attitudes Toward Telemedicine

Attitudes were assessed using seven statements rated on a 5-point Likert scale:

1 = Strongly disagree
2 = Disagree
3 = Neutral
4 = Agree
5 = Strongly agree

Items 1–6 were positively worded and scored directly. Item 7, “Telemedicine is a threat to current medical practice,” was negatively worded and reverse-coded.

The total attitude score ranged from 7 to 35. A higher score indicated a more positive attitude toward telemedicine.

Section 5: Perceptions Toward Telemedicine

Items 1–3 were assessed using a 5-point Likert scale and analyzed descriptively. No composite score was calculated.

Items 4 and 5 were assessed using three response options: Yes, No, and Maybe. These items were analyzed descriptively.
